# Association between Meteorological Factors and Outpatient Visits for Herpes Zoster in Hefei, China: A Time-Series Analysis

**DOI:** 10.3390/ijerph20032097

**Published:** 2023-01-23

**Authors:** Xiaojie Lv, Xinyu Fang, Tingting Qian, Yuyu Cai, Peng Gao, Haifeng Chen, Qing Wu, Jun Wu, Yinguang Fan, Dongqing Ye

**Affiliations:** 1 Department of Epidemiology and Biostatistics, School of Public Health, Anhui Medical University, Hefei 230032, China; 2 Inflammation and Immune Mediated Diseases Laboratory of Anhui Province, Hefei 230032, China; 3 Department of Epidemiology and Biostatistics, School of Public Health, Guangdong Medical University, Dongguan 523000, China

**Keywords:** herpes zoster, ambient temperature, relative humidity, time-series study, distributed lag nonlinear model

## Abstract

This study sought to investigate the relationship between meteorological factors and outpatient visits for herpes zoster. In this time-series analysis, we used data from two major hospitals in Hefei, collected between 2015 and 2019, to evaluate the impact of meteorological factors on the risk of herpes zoster. After controlling for confounders, we adopted a distributed lag nonlinear model to probe the relationship between meteorological factors and outpatient visits for herpes zoster. The analysis was stratified according to age (<40 years, ≥40 years) and sex (male, female). A total of 43,547 cases of herpes zoster were reported, and compared with the median value, a high temperature and high relative humidity had a significant risk effect on the incidence of herpes zoster. The maximum harmful effect of high temperature on herpes zoster occurred on the lag0 (RR: 1.027, 95% CI: 1.002–1.053) and further declined over the following days. The cumulative effect increased with the extension of lag days, and the cumulative RR was the largest on the sixth day of lag (RR1.031, 95% CI: 1.006–1.056) when the relative humidity was 85.7% (77.0% as the reference). The stratified analysis results reveal that females and the elderly (≥40 years) were more susceptible to temperature and relative humidity. This study shows that high-temperatures may lead to herpes zoster, indicating that those infected with varicella zoster virus need to take measures over the course of several days when not exposed to the best appropriate temperature conditions.

## 1. Introduction

Herpes zoster (HZ) is an infectious disease caused by the reactivation of the varicella-zoster virus (VZV), which can be concealed in the spinal ganglion or cranial nerves sensory ganglion for extensive periods of time [1,2]. HZ is almost always accompanied by postherpetic neuralgia, deprives the patients of normal sleep and a good quality of life, and even leads to certain disorders and depression [3,4]. The age-adjusted incidence rate of herpes zoster in the whole population in different countries around the world is (3–5)/1000 person years. The incidence of HZ in North America, Europe, and the Asia–Pacific region is similar and increases by 2.5–5.0% year by year [5]. According to a population-based cohort study, the incidence rate of HZ has more than quadrupled over the past 60 years, and vaccination has not affected the change in individual prevalence. The cause of this increase is unknown [6]. The incidence rate of HZ increased from 3.2 to 4.5 between 1997 and 2012. With the exception of children aged 0–9, its incidence has grown in all age groups [7]. It frequently happens to people with low immune function, such as those who receive chemotherapy, radiotherapy, or steroids, as well as those who receive disease-related immunosuppression as a result of HIV/AIDS, diabetes, or cancer. It is also common in people over 60 years of age because their immune systems weaken with age [1].

According to the United Nations Intergovernmental Panel on Climate Change’s (IPCC) sixth assessment report, the global average temperature in the years between 2011 and 2020 increased by approximately 1.09 °C compared to the period between 1850 and 1900. The likelihood and severity of extreme climate events including extreme precipitation and heat waves will rise in the future under the assumption of ongoing global warming. Climate change and global warming aggravate the health risks and disease burden of people [8]. Uncomfortable ambient temperatures are directly associated with an increased risk of heat stroke, cardiovascular illness, respiratory tract disease, and nervous system disease, according to some epidemiological research [9,10]. The observed seasonality indicates that meteorological factors might play a significant role in the epidemiology of HZ [11,12]. However, there is limited evidence on the associations between HZ and ambient temperature, which is one of the critical meteorological factors [13,14]. A study showed a significant correlation between the meteorological factors, especially temperature and wind speed, daily outpatient visits for skin diseases, the daily outpatient volume and temperature which have a positive and negative correlation, respectively, when the temperature is higher than the threshold [15]. A study based on a hospital that has nearly 7000 HZ patients in Shanghai used the generalized additive models (GAMs) with quasi-Poisson regression to examine the relationship between temperature and outpatient, and found that high temperature was a risk factor for HZ, and that if the daily average temperature rises by 1 °C, the number of outpatient visits of HZ will increase by 2.18% [16]. In South Korea, a nationwide large-scale study utilized GAM and generalized linear model (GLM) to focus on the relationship between ambient temperature and HZ, which showed that a higher ambient temperature resulted in an increased risk of HZ incidence rate [17]. Another ecological study suggested the relationship between the monthly incidence rate of HZ, and the analysis of the monthly average temperature found that high temperature is a hazard factor for HZ [18]. However, the majority of the research has mainly focused on understanding how temperature affects the frequency of HZ, and there is little if any data on how other meteorological conditions, including humidity, affect the number of patients with HZ.

As we all know that the effects of environmental factors on disease frequently act nonlinearly, cumulatively, and lagging, a distribution lag nonlinear model (DLNM) is suitable for this research [19]. Since 2010, DLNM has been used to investigate the effects of environmental factors on diseases; many studies have used the method to study dermatoses, but few have used it to study HZ. To fill this void, we used DLNM based on the quasi-Poisson generalized linear regression model to examine the relationship between the changes in meteorological factors and the number of HZ-related outpatient visits. We explained the short-term correlation between temperature and humidity and the number of hospital visits related to HZ, and we further evaluated the vulnerability differences among subgroups by level to determine the vulnerable population. Understanding the role of environmental factors in HZ will aid in the management of this complex disease and the reduction in disease burden on the health system.

## 2. Materials and Methods

### 2.1. Study Area

This study analyzed the data on outpatient visits for HZ from 2015 to 2019 in Hefei City, east China’s Anhui Province, with a typical subtropical monsoon climate and a resident population of 8.9 million as of 2019.

### 2.2. Case of Outpatient Visits for HZ

Daily outpatient visits for herpes zoster from 1 January 2015 to 31 December 2019 were obtained from the First Affiliated Hospital of Anhui Medical University and the First Affiliated Hospital of USTC. Additionally, the two 3A (first-class) hospitals have an advanced electronic data storage system that records all admitted patients. The information of patients mainly comprised the date of the outpatient, date of birth, age, residence address, sex, etc. The diagnosis of HZ was accomplished by dermatologists based on the patient’s symptoms, inquiries, reports, and findings on medical examination. Additionally, this research only uses cases with a clear and definite diagnosis. To avoid repeated inclusion, patients who met the diagnosis and were in two hospitals at the same time were treated twice. To ensure the integrity and accuracy of the collected data, data re-entry must take the form of double entry and third-party verification.

### 2.3. Environmental Variables

Daily meteorological data were acquired from the Hefei Meteorological Bureau, including daily mean temperature (MT), diurnal temperature range (DTR), wind speed (WS), relative humidity (RH), precipitation (PRE), sunshine hour (SSH), and barometric pressure (BP). Daily air pollutant data, including sulfur dioxide (SO_2_), particulate matter 10 (PM_10_), carbon monoxide (CO), particulate matter 2.5 (PM_2.5_), nitrogen dioxide (NO_2_), and ozone (O_3_) were collected from the Hefei Environmental Monitoring Center. The average values of daily air pollutant concentrations from these ten monitoring stations were taken as representative exposure levels for air pollutants in Hefei. The departments of environmental monitoring and meteorological management have a comprehensive monitoring and management system in place, and professional personnel are in charge of data entry and inspection. The data integrity is good, true, and reliable.

### 2.4. Statistical Analysis

During the study period, a descriptive analysis was performed for the information from outpatient visits for HZ, meteorological variables (daily mean temperature and RH) and air pollutants (PM_10_, NO_2_, SO_2_, PM_2.5_, and CO).

After adjusting for air pollutants, the long-term trend, day of the week and holiday, we used the Poisson generalized linear model (PGLM) and distributed lag nonlinear model (DLNM) to examine the relationships between meteorological variables and outpatient visits for HZ because these visits have a small probability of occurring [19]. DLNM can not only evaluate the impact of meteorological factors on public health but can also explore the relationship between predictive variables and outcomes and lag effects in a time-series study. To prevent multicollinearity, correlation analyses were performed to assess the relationships between air pollutants, meteorological factors, and the daily number of outpatient visits for herpes zoster, with a spearman correlation coefficient greater than 0.7 considered a high correlation. The final model is as follows:

(1) $$Y_{t}\sim quasipoisson\left(u_{t}\right)$$

(2) $$\mathrm{Log}{\left(\mu_{t}\right)}_{MT}=\alpha +\beta T_{t,l}+ns(RH_{t,l\;},3)+ns\left(Pollution\;confounders\;,\;3\right)+ns(Times_{t},6)+DOW_{t}+Holiday_{t}$$

(3) $$\mathrm{Log}{\left(\mu_{t}\right)}_{RH}=\alpha +\gamma RH_{t,l}+ns(T_{t,l\;},3)+ns\left(Pollution\;confounders\;,\;3\right)+ns(Times_{t},6)+DOW_{t}+Holiday_{t}$$

where *μ_t_* is the observed daily outpatient visits at day *t*; *α* represents the intercept of the model; *T_t,l_* is the matrixes produced by DLNM to model the mean temperature, *RH_t_*_,*l*_ is the matrixes produced by DLNM to model relative humidity, *l* is the lag day, *β* refers to the vector of *T_t_*, *γ* refers to the vector of *RH_t_*, and *ns* is the natural cubic spline; categorical variables including the day of the week (*Dow*) and holidays (*Holiday*) are also controlled. The optimal degrees of freedom (df) for meteorological factors, air pollutants, and time were chosen based on the Akaike Information Criterion (AIC) [19]. In order to guarantee that the results were robust, we also conducted sensitivity analyses by transforming the df values. We used a natural smooth function of calendar time with 6 degrees of freedom per year to control unmeasured long-term and seasonal trends, and a natural smooth function with 3 degrees of meteorological factors to control for its potential nonlinear confounding effects (9). The number of lag days corresponding to the lowest AIC value was what we need. Moreover, a further stratified analysis was performed by age (<40 years vs. ≥40 years) and sex (male vs. female) for ascertaining the susceptible population.

All statistical analyses were performed by R software (Version 4.0.5), and the “dlnm” and “spline” packages were used to calculate the relative risk (RR) and 95% confidence interval (CI), with the 75th percentiles and 25th percentiles of daily mean temperature and relative humidity, respectively, compared with 17.6 °C (median value of mean temperature) and 77% (median value of relative humidity). The statistical tests were two-sided, and effects with a *p* value of less than 0.05 were considered statistically significant.

## 3. Results

### 3.1. Data Description

From 1 January 2015 to 31 December 2019, the medical information system from the First Affiliated Hospital of Anhui Medical University and the First Affiliated Hospital of USTC recorded a total of 43,547 HZ patients, with an average of approximately 23.85 cases per day. In addition, females accounted for 54.43% and patients aged ≥40 years accounted for 74.56%. The daily mean temperature, DTR, and RH were 16.85 °C, 8.39 °C, and 76.51%, respectively. All the relevant records are shown in Table 1.

**Figure 1:**
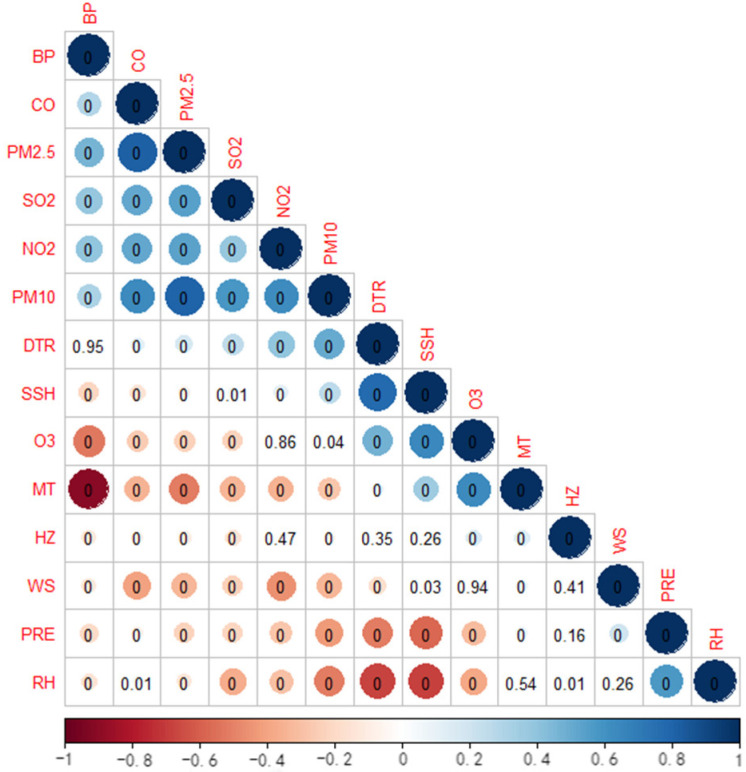
Spearman’s correlation coefficients between weather conditions and major pollution.

Figure 1 and Table S1 reflect the correlation between meteorological parameters and daily HZ outpatients. The HZ visits were positively correlated with MT, RH, and O_3_ (*p* < 0.05), and were negatively correlated with BP, SO_2_, PM_10_, PM_2.5_, and CO (*p* < 0.05). However, the correlations between HZ and PRE, SSH, WS, DTR, and NO_2_ had no statistical significance (*p* > 0.05). MT was strongly correlated with atmospheric pressure (*r* = 0.909), and positively correlated with DTR (*p* < 0.001), while the mean temperature was not correlated with RH (*p* = 0.54). Furthermore, there was a nonlinear relationship between the MT and HZ outpatient visits. There were a number of herpes zoster outpatient visits, and the RH, daily mean temperature, and DTR in Hefei City from 2015 to 2019 displayed a seasonal pattern (Figure S1).

### 3.2. Lagged Effects of Temperature and Relative Humidity

**Figure 2:**
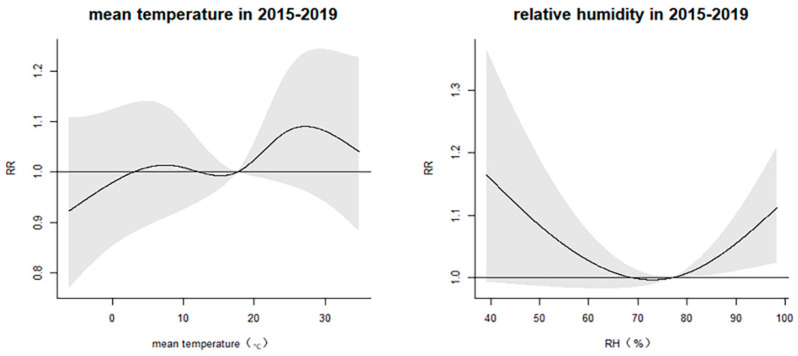
The overall exposure–response association curve between mean temperature and relative humidity and daily outpatient visits for HZ.

Figure 2 shows that the exposure–response curve of temperature on HZ was presented as an “M” curve. When the temperature was above 17.6 °C, the temperature has an obvious positive effect on the incidence of HZ. The RRs of relative humidity on HZ are shown as an “V” curve. Additionally, the RRs that increased with an RH above 77% were significant.

**Figure 3:**
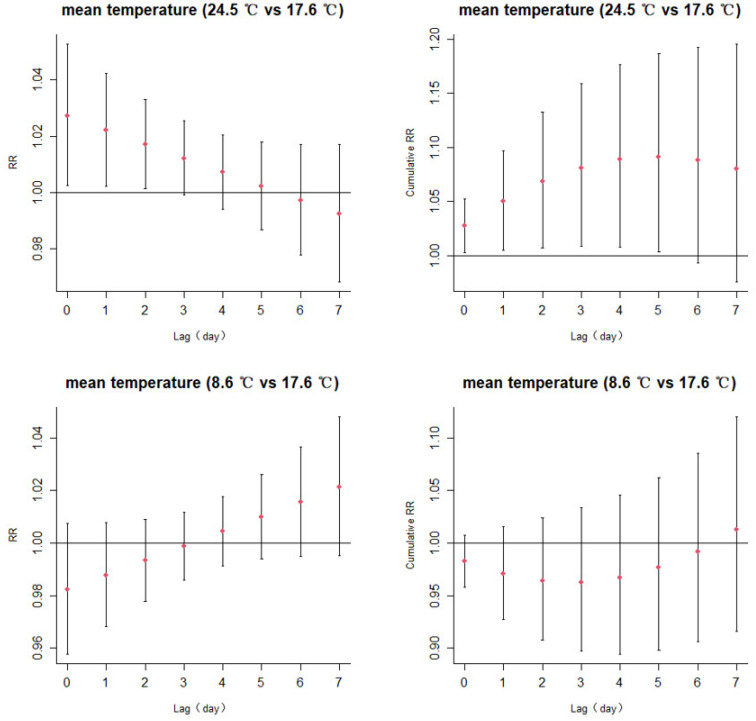
Lag-specific relative risks (95%CI) and cumulative risks (95%CI) in outpatient visits for herpes zoster with high (75th percentile 24.5 °C) and low (25th percentile 8.6 °C) levels of the mean temperature in the model.

Despite extreme conditions, meteorological factors have different effects on the incidence of a herpes zoster on different lag days. Figure 3 shows the lag effects for a mean temperature of the 75th percentile (24.5 °C) and 25th percentile (8.6 °C) with the reference of 17.6 °C (median value of mean temperature). The odds of outpatient visits for HZ were increased by high temperatures (lag0, relative risk (RR):1.027, 95% CI: 1.002–1.053; Table S2), and the RRs were highest on the lag0 and persisted for 3 days in odd-day lag effects. Similarly, the maximum cumulative lag effect of high temperature reached statistical significance when the lag day was 0–5 days (RR = 1.091, 95% CI = 1.003–1.187) (Table S2). The relationship between low-temperature and HZ appeared a protective effect, but the difference was not statistically significant.

**Figure 4:**
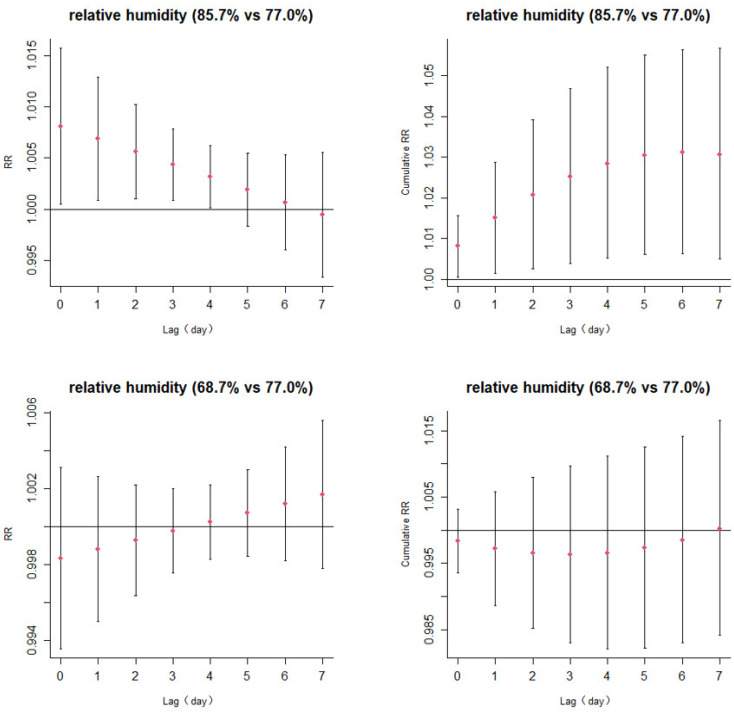
Lag-specific relative risks (95%CI) and cumulative risks (95%CI) in outpatient visits for the herpes zoster with high (75th percentile 85.7%) and low (25th percentile 68.7%) levels of relative humidity in the model.

Figure 4 shows that the high relative humidity was positively correlated with HZ, and the single-lag effects from 0 to 4 days were statistically significant. The cumulative RRs gradually increased with the prolongation of the lag time, and reached a maximum of 1.031 (95% CI: 1.006 – 1.056) (Table S2) on the lag6 days.

### 3.3. Subgroup Analyses by Individual Characteristics

The results of the stratification analysis, on account of the different sexes and age groups, are exhibited in Table 2 and Table 3. The effect was most pronounced among females and the elderly, which were the sensitive groups in terms of temperature and relative humidity. For high temperature, the subgroup analyses showed that the influence on the ≥40 years group had a harmful effect, and the highest RR at lag1 was 1.023 (95% CI = 1.001–1.047). Additionally, the harmful effects caused by low temperature appeared on the lag5 and disappeared on the lag7 in the female group. Compared with the low relative humidity, under the high relative humidity, females and the elderly were significantly affected relative humidity (85.7%) by the single-day lag effect, whilst the maximum RR value was 1.005 (95% CI: 1.000–1.009) and 1.011 (95% CI: 1.003–1.020), respectively.

### 3.4. Sensitivity Analysis

The main findings did not significantly change in the sensitivity analyses, indicating that the findings were robust. As these were sensitivity analyses, we varied the degree of freedom of the meteorological factors (3–5 dfs), pollution confounders (3–5 dfs), and time (6–8 dfs per year) (Figures S2–S5). When the degree of freedom of the variable is changed, the difference is subtle when compared to the original model results, and the results are statistically significant. It is demonstrated that pollutants, other meteorological factors, and time variables have no discernible impact on the results of the model established in this study, implying that the model is stable and the fitting results are ideal.

## 4. Discussion

In this study, we used DLNM to evaluate the correlation between meteorological factors and HZ outpatient visits in Hefei, China, during the period 2015–2019. We found that the association between temperature and relative humidity significantly increased the risk HZ-related outpatient visits.

Higher temperature was related to the increased RRs of HZ outpatient visits. Females and the elderly are vulnerable groups. Some other previous studies have already supported these conclusions. According to Berlinberg’s research, HZ may be seasonal and reach its peak incidence in summer [20]. In addition, a large-scale survey stated that the incidence of HZ was higher in spring and summer than in winter [13,21]. Little is known about the underlying mechanisms of seasonal variations in HZ morbidity. When VZV reactivates in the human parasitifer, it effectively duplicates in the skin and produces high titers of infectious virus in the skin pathologies [22]. In fact, a spectrum of immunosuppression plays an important role in the reactivation of VZV [23]. Some mechanisms may interpret why the high level of temperature increases the number of outpatients for HZ. Firstly, as we all know, VZV’s function cannot be ignored with regard to the occurrence and development of HZ [24]. Dopico et al. [25] stated that seasonal gene expression was selected in the process of evolution, such that VZV co-evolves with other infectious microorganisms and the interspecific competition is more intense in winter. Then, the immune response to VZV will be reduced in summer, which may induce greater susceptibility in people to the reactivation of latent VZV infection. Additionally, previous studies have suggested that ultraviolet radiation (UVR) may contribute to seasonality [26]. UVR-mediated immunosuppression is related to the reactivation of the lurking infection of VZV, and high UVR is usually accompanied in summer, which contributes to the ascendance of HZ morbidity [27,28]. MacGillivray et al. showed that environmental factors play an important role in modulating the immune system [29]. Consequently, it seems plausible that the ambient temperature transformation may disturb the attack of HZ, which is an immunologically related disease.

The exposure–response curve between the relative humidity and HZ showed a “U” shape. With the increase in RH, a relative risk decreased at first and then increased. When the humidity is greater than 77%, RRs are above 1.000, which is statistically significant, and indicates that a high relative humidity is a harmful factor for HZ. Moreover, in terms of lag effect, when the relative humidity is 85.7%, the accumulated RR is the largest at lag6. Denda et al. [30] speculated that the low relative humidity stimulated the lamellar body secretory system, promoting barrier repair in the skin, whereas high relative humidity may delay barrier repair. According to this study, we may explain the mechanism of the effect of relative humidity exposure on HZ outpatient visits.

As one of the research purposes was to determine susceptible populations, stratified analysis suggested that sex and age subgroups were adopted. For both temperature or relative humidity, a statistical association was observed among the elderly and females. For the sex subgroup, a higher overall HZ incidence was previously reported in females [13,17,31]. Fleming et al. [31] investigated the incidence of shingles and found female excesses in all age groups of HZ, which is consistent with this study. This may be related the fact that the females were more frequently in contact with children who were infected with chicken pox than males [32]. For the age subgroup, the temperature had a greater influence on the elderly patients with HZ. Theoretically, the elderly undergo immunosenescence, which is a major cause of increased VZV infection and reactivation [33].

There were some superiorities in our research. Previous studies adopted traditional and single models, such as the general additive model and general linear model, which only considered the effect in a specific period and ignored the lag effects of temperature. DLNM was right for describing nonlinear and hysteresis effects. Meanwhile, several limitations should be acknowledged. First, we only used two hospitals in one city, and the result may be not representative. Second, the study was an ecological study, which had ecological fallacies and was unavailable to analyze the causal relationship between HZ and meteorological factors. Upon this condition, our study may encourage other populations from different countries of the world to conduct future research. Finally, the factors that contributed to the onset and treatment of herpes zoster are relatively complex, and some confounding factors, such as air pollutants, patients’ immune levels, genetic factors, patients’ economic status, and other related factors, cannot be controlled. In addition, further study should be focused on molecular chemistry and biochemistry to detect the relationship between meteorological factors and HZ.

## 5. Conclusions

In a word, our study provides quantitative proof of the relevance of ambient temperature and relative humidity to HZ outpatient visits, indicating that both the high temperature and high RH may increase the risk of HZ over five years in subtropical cities and that the populations of females aged ≥40 years were vulnerable people. These findings will help local hospitals arrange and manage the visits of HZ patients before the weather changes and be act as valuable guides for conducting public health interventions.

## Figures and Tables

**Table 1:** Summary statistics of outpatient visits for HZ and meteorological variables in Hefei, China, during the period 2015–2019.

Variables	Sum	Mean (SD)	Minimum	P_25_	P_50_	P_75_	Maximum
Outpatient visits	43,547	23.85 (6.79)	0.00	19.00	24.00	28.00	50.00
Male	19,846	10.87 (3.91)	0.00	8.00	11.00	13.00	27.00
Female	23,701	12.89 (4.54)	0.00	10.00	13.00	16.00	31.00
40 years	11,078	6.07 (2.69)	0.00	4.00	6.00	8.00	17.00
≥40 years	32,469	17.78 (5.83)	0.00	14.00	18.00	21.00	42.00
Daily mean Temperature (°C)		16.85 (9.21)	−6.20	8.60	17.60	24.50	34.80
DTR (°C)		8.39 (3.77)	0.80	5.50	8.30	11.10	19.80
Relative humidity (%)		76.51 (11.98)	39.20	68.70	77.00	85.70	98.30

**Table 2:** The relative risk of the 75th percentile of ambient temperature and relative humidity on HZ outpatients in Hefei.

	lag0	lag1	lag2	lag3	lag4	lag5	lag6	lag7
MT								
Female	1.023 (0.990,1.057)	1.020 (0.993,1.046)	1.016 (0.995,1.037)	1.012 (0.995,1.030)	1.009 (0.992,1.026)	1.005 (0.985,1.026)	1.002 (0.976,1.028)	0.998 (0.966,1.031)
Male	1.032 (0.997,1.068)	1.025 (0.997,1.054)	1.018 (0.996,1.041)	1.012 (0.993,1.031)	1.005 (0.987,1.024)	0.998 (0.977,1.021)	0.992 (0.965,1.020)	0.985 (0.952,1.020)
0–40 years	1.032 (0.986,1.081)	1.019 (0.982,1.058)	1.007 (0.978,1.037)	0.994 (0.970,1.019)	0.982 (0.958,1.006)	0.969 (0.942,0.998) *	0.957 (0.923,0.993) *	0.945 (0.903,0.990) *
≥40 years	1.026 (0.997,1.055)	1.023 (1.001,1.047) *	1.021 (1.003,1.039) *	1.019 (1.004,1.034) *	1.016 (1.001,1.032) *	1.014 (0.996,1.032)	1.012 (0.989,1.035)	1.009 (0.981,1.038)
RH								
Female	1.008 (0.998,1.018)	1.007 (0.999,1.015)	1.006 (1.000,1.012)	1.005 (1.000,1.009) *	1.004 (1.000,1.008)	1.003 (0.998,1.007)	1.002 (0.995,1.008)	1.000 (0.992,1.009)
Male	1.008 (0.997,1.019)	1.007 (0.998,1.015)	1.005 (0.999,1.012)	1.004 (0.999,1.009)	1.002 (0.998,1.007)	1.001 (0.996,1.006)	1.000 (0.993,1.006)	0.998 (0.990,1.007)
0–40 years	0.998 (0.984,1.013)	1.000 (0.988,1.011)	1.001 (0.992,1.010)	1.002 (0.996,1.009)	1.003 (0.998,1.009)	1.005 (0.998,1.011)	1.006 (0.997,1.015)	1.007 (0.996,1.019)
≥40 years	1.011 (1.003,1.020) *	1.009 (1.002,1.016) *	1.007 (1.002,1.013) *	1.005 (1.001,1.009) *	1.003 (0.999,1.006)	1.001 (0.997,1.005)	0.999 (0.993,1.004)	0.997 (0.990,1.004)

* *p* < 0.05.

**Table 3:** Relative risk of the 25th percentile of ambient temperature and relative humidity on HZ outpatients in Hefei.

	lag0	lag1	lag2	lag3	lag4	lag5	lag6	lag7
MT								
Female	0.975 (0.943,1.009)	0.984 (0.958,1.011)	0.994 (0.973,1.015)	1.003 (0.986,1.020)	1.012 (0.995,1.030)	1.022 (1.000,1.044) *	1.032 (1.004,1.060) *	1.041 (1.006,1.078) *
Male	0.991 (0.956,1.026)	0.992 (0.964,1.020)	0.993 (0.972,1.015)	0.994 (0.976,1.012)	0.995 (0.977,1.014)	0.997 (0.975,1.019)	0.998 (0.970,1.027)	0.999 (0.964,1.036)
0–40 years	0.983 (0.937,1.032)	0.992 (0.954,1.030)	1.000 (0.971,1.030)	1.008 (0.984,1.033)	1.017 (0.992,1.042)	1.025 (0.995,1.057)	1.034 (0.994,1.075)	1.043 (0.993,1.095)
≥40 years	0.982 (0.954,1.011)	0.986 (0.964,1.009)	0.991 (0.973,1.009)	0.996 (0.981,1.010)	1.000 (0.985,1.015)	1.005 (0.987,1.023)	1.009 (0.986,1.033)	1.014 (0.984,1.045)
RH								
Female	1.000 (0.993,1.006)	1.000 (0.995,1.005)	1.000 (0.996,1.004)	1.001 (0.998,1.004)	1.001 (0.998,1.004)	1.001 (0.998,1.004)	1.002 (0.998,1.006)	1.002 (0.997,1.007)
Male	0.997 (0.990,1.004)	0.997 (0.992,1.003)	0.998 (0.994,1.002)	0.999 (0.996,1.002)	0.999 (0.997,1.002)	1.000 (0.997,1.003)	1.001 (0.997,1.005)	1.002 (0.996,1.007)
0–40 years	0.997 (0.988,1.006)	0.998 (0.991,1.005)	0.999 (0.993,1.004)	0.999 (0.995,1.004)	1.000 (0.997,1.004)	1.001 (0.997,1.005)	1.002 (0.996,1.007)	1.003 (0.995,1.010)
≥40 years	0.999 (0.993,1.004)	0.999 (0.995,1.003)	1.000 (0.996,1.003)	1.000 (0.997,1.002)	1.000 (0.998,1.003)	1.001 (0.998,1.003)	1.001 (0.998,1.005)	1.001 (0.997,1.006)

* *p* < 0.05.

## Data Availability

The available data on meteorological variables, air pollutants, and outpatient visits were used under the license for the current study, and are therefore not publicly available.

## Supplementary Materials

The following supporting information can be downloaded at: https://www.mdpi.com/article/10.3390/ijerph20032097/s1, Table S1: Spearman’s correlation coefficients between weather conditions and major pollution; Table S2: Lag-specific relative risks (95%CI) and cumulative risks (95%CI) in outpatient visits for herpes zoster with high (75th percentile) and low (25th percentile) levels of MT and RH in the model; Figure S1: Time series of HZ, mean temperature, DTR, and relative humidity in Hefei, China, from 2015 to 2019; Figure S2: The single-day effects of association between high level mean temperature (75th percentile 24.5 °C) and outpatient visits for HZ when varying the degrees of freedom (3–5 dfs) for meteorological variables and the df (7–9 dfs/year) for time; Figure S3: The single-day effects of association between low level mean temperature (25th percentile 8.6 °C) and outpatient visits for HZ when varying the degrees of freedom (3–5 dfs) for meteorological variables and the df (6–8 dfs/year) for time; Figure S4: The single-day effects of association between high level relative humidity (75th percentile 85.7%) and outpatient visits for HZ when varying the degrees of freedom (3–5 dfs) for meteorological variables and the df (6–8 dfs/year) for time; Figure S5: The single-day effects of association between low level relative humidity (25th percentile 68.7%) and outpatient visits for HZ when varying the degrees of freedom (3–5 dfs) for meteorological variables and the df (6–8 dfs/year) for time.

## References

[1] Schmader K. (2018). Herpes Zoster. Ann. Intern. Med..

[2] Yu Y.-H., Lin Y., Sun P.-J. (2019). Segmental Zoster Abdominal Paresis Mimicking an Abdominal Hernia. Medicine.

[3] Oster G., Harding G., Dukes E., Edelsberg J., Cleary P.D. (2005). Pain, Medication Use, and Health-Related Quality of Life in Older Persons with Postherpetic Neuralgia: Results from a Population-Based Survey. J. Pain.

[4] Ding X., Jiang W., Jiang J., Hu Y., Wu Y., Li Y., Zhao D., Yin D. (2021). The study on the relationship between herpes zoster and depression on Health Big Data-real word research examples. Chin. J. Dis. Control Prev..

[5] Kawai K., Gebremeskel B.G., Acosta C.J. (2014). Systematic Review of Incidence and Complications of Herpes Zoster: Towards a Global Perspective. BMJ Open.

[6] Kawai K., Yawn B.P., Wollan P., Harpaz R. (2016). Increasing Incidence of Herpes Zoster Over a 60-Year Period from a Population-Based Study. Clin. Infect. Dis..

[7] Marra F., Chong M., Najafzadeh M. (2016). Increasing Incidence Associated with Herpes Zoster Infection in British Columbia, Canada. BMC Infect. Dis..

[8] Romanello M., McGushin A., Di Napoli C., Drummond P., Hughes N., Jamart L., Kennard H., Lampard P., Solano Rodriguez B., Arnell N. (2021). The 2021 Report of the Lancet Countdown on Health and Climate Change: Code Red for a Healthy Future. Lancet.

[9] Chen R., Yin P., Wang L., Liu C., Niu Y., Wang W., Jiang Y., Liu Y., Liu J., Qi J. (2018). Association between Ambient Temperature and Mortality Risk and Burden: Time Series Study in 272 Main Chinese Cities. BMJ.

[10] Wang Y., Wang A., Zhai J., Tao H., Jiang T., Su B., Yang J., Wang G., Liu Q., Gao C. (2019). Tens of Thousands Additional Deaths Annually in Cities of China between 1.5 °C and 2.0 °C Warming. Nat. Commun..

[11] Shi X.M. (2021). Promoting research on air pollution, climate change and population health under the goal of carbon neutrality and at the peak carbon dioxide emissions. Chin. J. Dis. Control Prev..

[12] Chen Y., Leng R.X., Pan H.F. (2021). Research advance on the role of meteorolgical factors in inflammatory immune diseases. Chin. J. Dis. Control Prev..

[13] Toyama N., Shiraki K., Society of the Miyazaki Prefecture Dermatologists (2009). Epidemiology of Herpes Zoster and Its Relationship to Varicella in Japan: A 10-Year Survey of 48,388 Herpes Zoster Cases in Miyazaki Prefecture. J. Med. Virol..

[14] Korostil I.A., Regan D.G. (2016). Varicella-Zoster Virus in Perth, Western Australia: Seasonality and Reactivation. PLoS ONE.

[15] Zhang W., Zhang Y.-J., Shen X.-P., Ning G.-C., Wei Y.-J., Zeng W., Yu D.-H., Lu H.-G. (2021). Effects of Meteorological Factors on Daily Outpatient Visits for Skin Diseases: A Time Series Study in a Chinese Population. Chin. Med. J..

[16] Yang Y., Chen R., Xu J., Li Q., Xu X., Ha S., Song W., Tan J., Xu F., Kan H. (2015). The Effects of Ambient Temperature on Outpatient Visits for Varicella and Herpes Zoster in Shanghai, China: A Time-Series Study. J. Am. Acad. Dermatol..

[17] Choi Y.-J., Lim Y.-H., Lee K.-S., Hong Y.-C. (2019). Elevation of Ambient Temperature Is Associated with an Increased Risk of Herpes Zoster: A Time-Series Analysis. Sci. Rep..

[18] Lai S.-W., Liao K.-F., Kuo Y.-H., Lin C.-L., Liu C.-S., Hwang B.-F., Lai Y.-J. (2021). The Impacts of Ambient Temperature and Ultraviolet Radiation on the Incidence of Herpes Zoster: An Ecological Study in Taiwan. Int. J. Clin. Pract..

[19] Gasparrini A., Armstrong B., Kenward M.G. (2010). Distributed Lag Non-Linear Models. Stat. Med..

[20] Berlinberg E.J., Kim E., Deiner M.S., Patterson C., Porco T.C., Acharya N.R. (2020). Seasonality of Herpes Zoster and Herpes Zoster Ophthalmicus. J. Clin. Virol..

[21] Jung H.S., Kang J.K., Yoo S.H. (2015). Epidemiological Study on the Incidence of Herpes Zoster in Nearby Cheonan. Korean J. Pain.

[22] Abendroth A., Arvin A.M. (2001). Immune Evasion as a Pathogenic Mechanism of Varicella Zoster Virus. Semin. Immunol..

[23] Gershon A.A., Breuer J., Cohen J.I., Cohrs R.J., Gershon M.D., Gilden D., Grose C., Hambleton S., Kennedy P.G.E., Oxman M.N. (2015). Varicella Zoster Virus Infection. Nat. Rev. Dis. Prim..

[24] Reichelt M., Zerboni L., Arvin A.M. (2008). Mechanisms of Varicella-Zoster Virus Neuropathogenesis in Human Dorsal Root Ganglia. J. Virol..

[25] Dopico X.C., Evangelou M., Ferreira R.C., Guo H., Pekalski M.L., Smyth D.J., Cooper N., Burren O.S., Fulford A.J., Hennig B.J. (2015). Widespread Seasonal Gene Expression Reveals Annual Differences in Human Immunity and Physiology. Nat. Commun..

[26] Moyal D.D., Fourtanier A.M. (2008). Broad-Spectrum Sunscreens Provide Better Protection from Solar Ultraviolet-Simulated Radiation and Natural Sunlight-Induced Immunosuppression in Human Beings. J. Am. Acad. Dermatol..

[27] Fatahzadeh M., Schwartz R.A. (2007). Human Herpes Simplex Virus Infections: Epidemiology, Pathogenesis, Symptomatology, Diagnosis, and Management. J. Am. Acad. Dermatol..

[28] Freeman R.G., Knox J.M. (1964). Influence of Temperature on Ultraviolet Injury. Arch. Dermatol..

[29] MacGillivray D.M., Kollmann T.R. (2014). The Role of Environmental Factors in Modulating Immune Responses in Early Life. Front. Immunol..

[30] Denda M., Sato J., Masuda Y., Tsuchiya T., Koyama J., Kuramoto M., Elias P.M., Feingold K.R. (1998). Exposure to a Dry Environment Enhances Epidermal Permeability Barrier Function. J. Investig. Dermatol..

[31] Fleming D.M., Cross K.W., Cobb W.A., Chapman R.S. (2004). Gender Difference in the Incidence of Shingles. Epidemiol. Infect..

[32] Opstelten W., Van Essen G.A., Schellevis F., Verheij T.J.M., Moons K.G.M. (2006). Gender as an Independent Risk Factor for Herpes Zoster: A Population-Based Prospective Study. Ann. Epidemiol..

[33] Ho T.-Y., Chung C.-H., Shen Y.-P., Chen L.-C., Chien W.-C., Wu Y.-T. (2019). Herpes Zoster Increased Risk of Neuralgic Amyotrophy: A Retrospective, Population-Based Matched Cohort Study. J. Neurovirol..

