# Supplementary material for: Association between Meteorological Factors and Outpatient Visits for Herpes Zoster in Hefei, China: A Time-Series Analysis
Source: Int J Environ Res Public Health. 2023 Jan 23;20(3):2097. doi: 10.3390/ijerph20032097 (PMC9915272; doi:10.3390/ijerph20032097)
Supplement: Supplementary file 1 [file ijerph-20-02097-s001.zip › ijerph-2079492-supplementary.pdf]

Association between meteorological factors and outpatient visits for herpes zoster in Hefei, China: a time-series analysis

Table S1. Spearman's correlation coefficients between weather conditions and major pollution.

	HZ	MT	RH	BP	PRE	SSH	WS	DTR	O3	SO2	NO2	PM10	PM2.5
MT	0.135*												
RH	0.061*	0.014											
BP	-0.122*	-0.909*	-0.156*										
PRE	0.033	0.079*	0.579*	-0.199*									
SSH	0.026	0.356*	-0.681*	-0.217*	-0.579*								
WS	-0.019	0.085*	-0.027	-0.125*	0.201*	-0.052*							
DTR	-0.022	0.083*	-0.679*	-0.002	-0.511*	0.777*	-0.178*						
O_3	0.156*	0.633*	-0.388*	0.525*	-0.310*	0.647*	0.002	0.478*					
SO_2	-0.156*	-0.335*	-0.365*	0.386*	-0.219*	0.063*	-0.230*	0.256*	-0.224*				
NO_2	-0.017	-0.348*	-0.297*	0.395*	-0.274*	0.117*	-0.456*	0.390*	0.004	0.382*			
PM_{10}	-0.075*	-0.276*	-0.512*	0.317*	-0.428*	0.261*	-0.336*	0.506*	0.047*	0.581*	0.626*		
$PM_{2.5}$	-0.095*	-0.513*	-0.133*	0.469*	-0.225*	-0.088*	-0.339*	0.177*	-0.238*	0.549*	0.532*	0.806*	
CO	-0.080*	-0.344*	0.058*	0.296*	-0.080*	-0.153*	-0.409*	0.114*	-0.258*	0.512*	0.511*	0.639*	0.812*

*P<0.05.

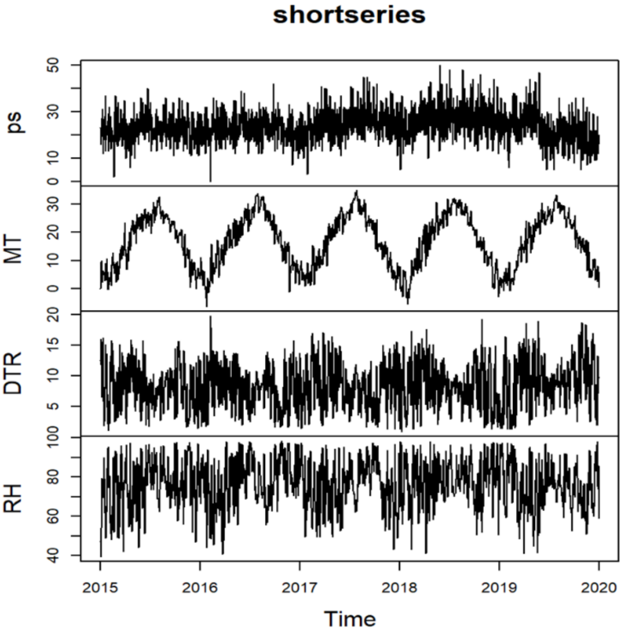

Figure S1. Time series of HZ, mean temperature, DTR, and relative humidity in Hefei, China, from 2015 to 2019.

Table S2. Lag-specific relative risks (95%CI) and cumulative risks (95%CI) in outpatient visits for herpes zoster with high(75th percentile) and low(25th percentile) levels of MT and RH in the model.

	lag0	lag1	lag2	lag3	lag4	lag5	lag6	lag7
High MT								
RR	1.027<br>(1.002,1.05)	1.022<br>(1.002,1.042)	1.017<br>(1.001,1.033)	1.012<br>(0.999,1.025)	1.007<br>(0.994,1.020)	1.002<br>(0.987,1.018)	0.997<br>(0.978,1.017)	0.992<br>(0.968,1.017)
cumRR	1.027<br>(1.002,1.053)	1.050<br>(1.005,1.097)	1.068<br>(1.007,1.133)	1.081<br>(1.008,1.159)	1.089<br>(1.007,1.177)	1.091<br>(1.003,1.187)	1.088<br>(0.993,1.192)	1.080<br>(0.976,1.196)
Low MT								

RR	0.982 (0.958,1.008)	0.988 (0.968,1.008)	0.993 (0.978,1.009)	0.999 (0.986,1.012)	1.004 (0.991,1.018)	1.010 (0.994,1.026)	1.016 (0.995,1.037)	1.021 (0.995,1.048)
cumRR	0.982 (0.958,1.008)	0.970 (0.927,1.015)	0.964 (0.908,1.024)	0.963 (0.897,1.033)	0.967 (0.894,1.046)	0.977 (0.898,1.062)	0.992 (0.906,1.086)	1.013 (0.916,1.120)
High RH								
RR	1.008 (1.001,1.016)	1.007 (1.001,1.013)	1.006 (1.001,1.010)	1.004 (1.001,1.008)	1.003 (1.000.1.006)	1.002 (0.998,1.005)	1.001 (0.996,1.005)	0.999 (0.993,1.006)
cumRR	1.008 (1.001,1.016)	1.015 (1.001,1.029)	1.021 (1.003,1.039)	1.025 (1.004,1.047)	1.028 (1.005,1.052)	1.030 (1.006,1.055)	1.031 (1.006,1.056)	1.031 (1.005,1.057)
Low RH								
RR	0.998 (0.994,1.003)	0.999 (0.995,1.003)	0.999 (0.996,1.002)	1.000 (0.998,1.002)	1.000 (0.998,1.002)	1.001 (0.998,1.003)	1.001 (0.998,1.004)	1.002 (0.998,1.006)
cumRR	0.998 (0.994,1.003)	0.997 (0.989,1.006)	0.996 (0.985,1.008)	0.996 (0.983,1.010)	0.997 (0.982,1.011)	0.997 (0.982,1.013)	0.999 (0.983,1.014)	1.000 (0.984,1.017)

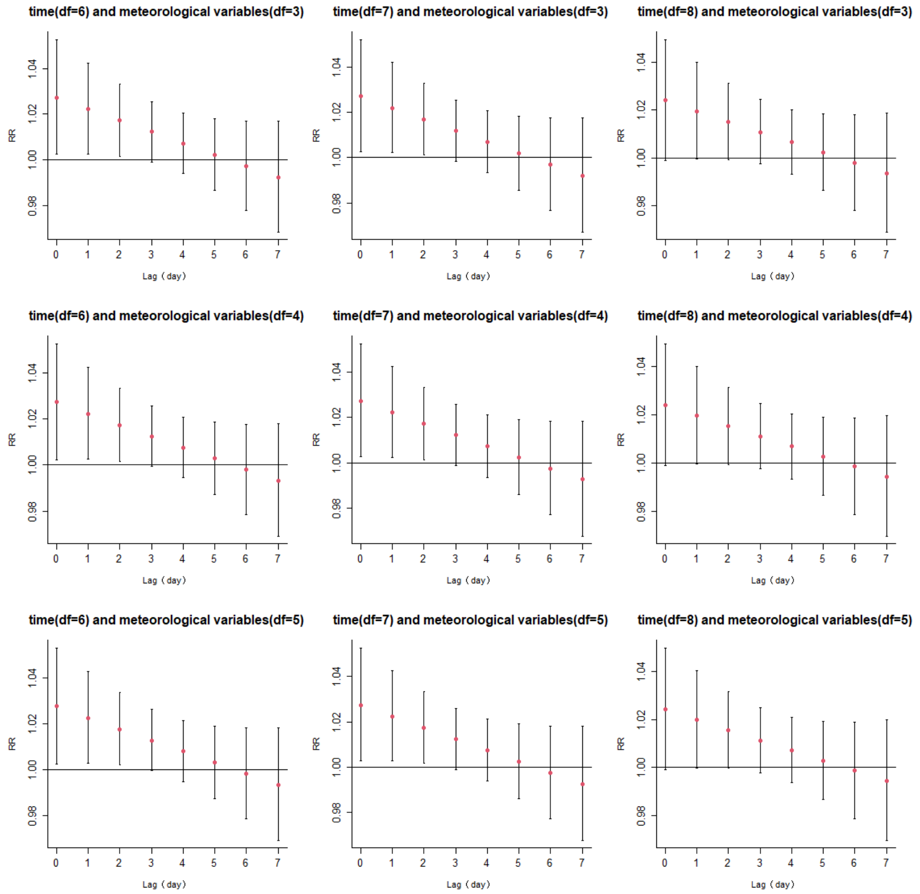

Figure S2. The single-day effects of association between high level mean temperature (75th percentile 24.5°C) and outpatient visits for HZ when varying the degrees of freedom (3-5dfs) for meteorological variables and the df (7-9 dfs/year) for time.

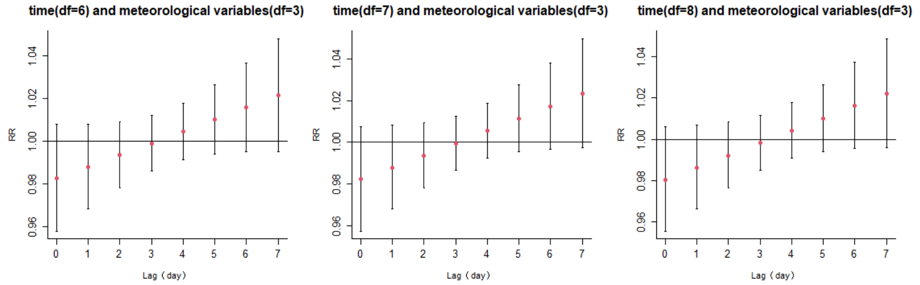

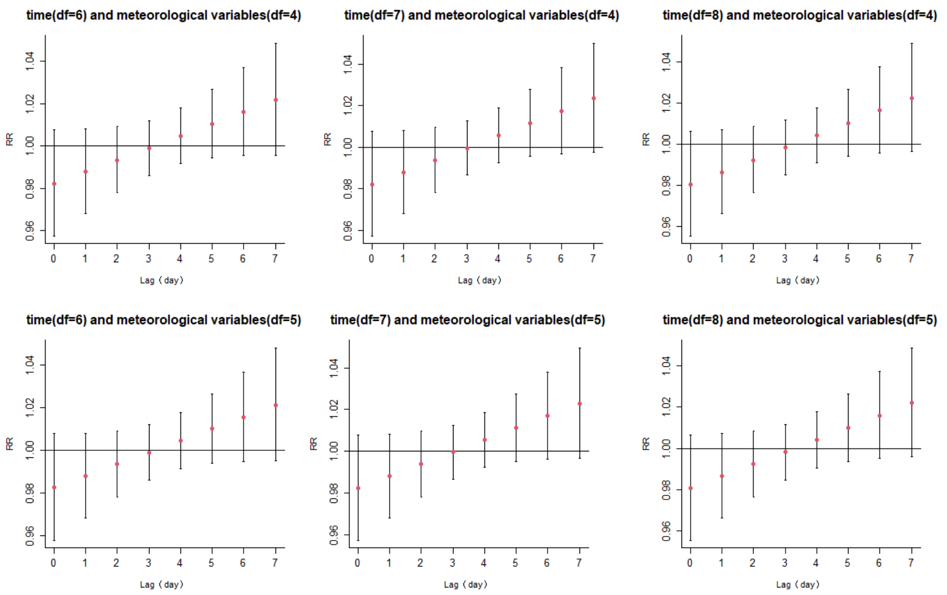

Figure S3. The single-day effects of association between low level mean temperature (25th percentile 8.6°C) and outpatient visits for HZ when varying the degrees of freedom (3-5dfs) for meteorological variables and the df (6-8 dfs/year) for time.

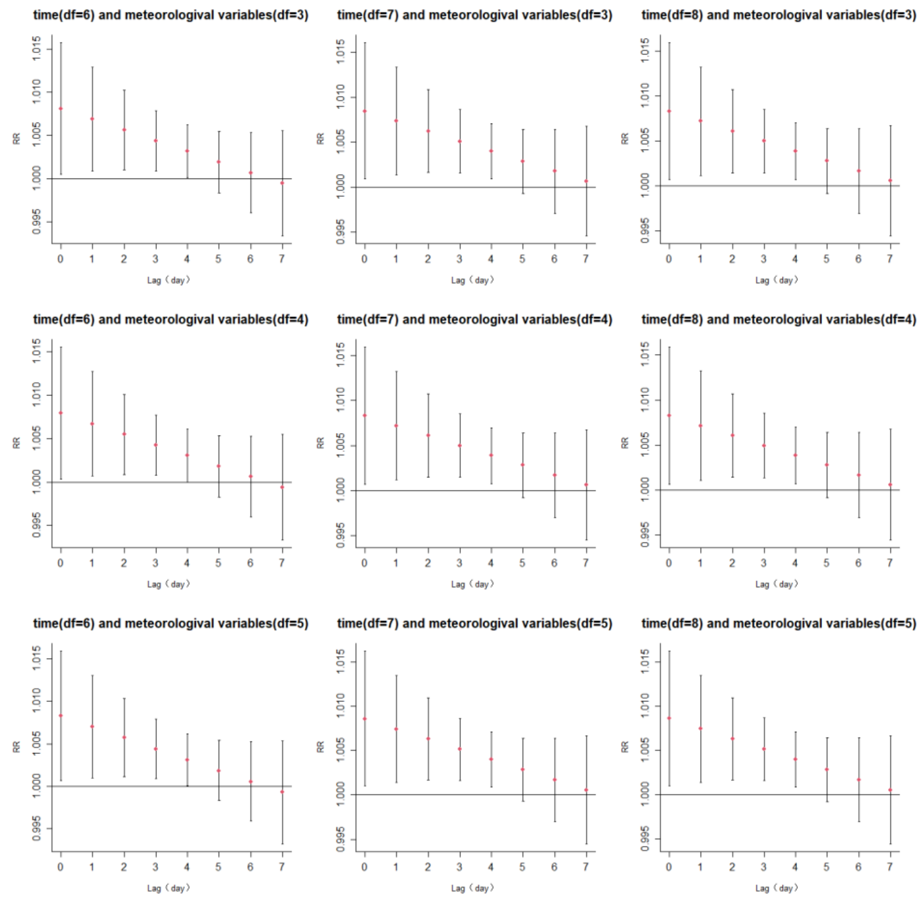

Figure S4. The single-day effects of association between high level relative humidity (75th percentile 85.7%) and outpatient visits for HZ when varying the degrees of freedom (3-5dfs) for meteorological variables and the df (6-8 dfs/year) for time.

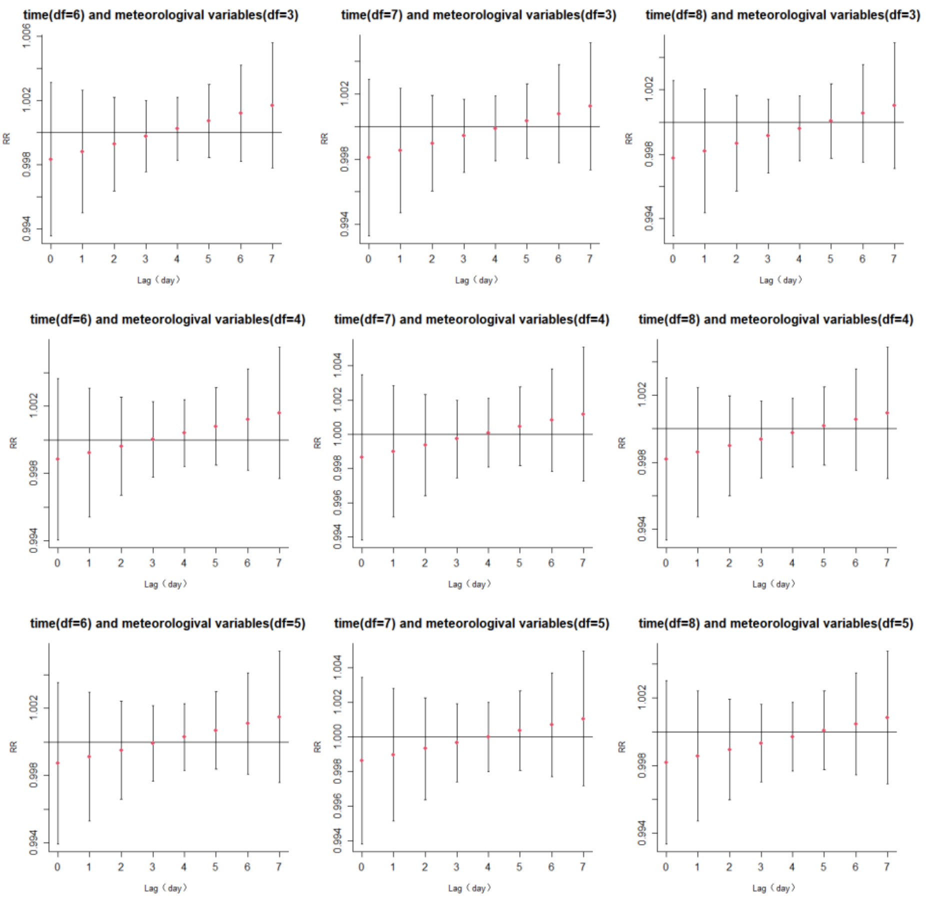

Figure S5. The single-day effects of association between low level relative humidity (25th percentile 68.7%) and outpatient visits for HZ when varying the degrees of freedom (3-5dfs) for meteorological variables and the df (6-8 dfs/year) for time.
